# What Can CISS Teach Us about Electron Transfer?

**DOI:** 10.1021/acs.jpclett.4c02617

**Published:** 2024-10-27

**Authors:** Ron Naaman, David H. Waldeck

**Affiliations:** † Department of Chemical and Biological Physics, Weizmann Institute of Science, 76100 Rehovot, Israel; ‡ Department of Chemistry, University of Pittsburgh, Pittsburgh, Pennsylvania 15260 United States

## Abstract

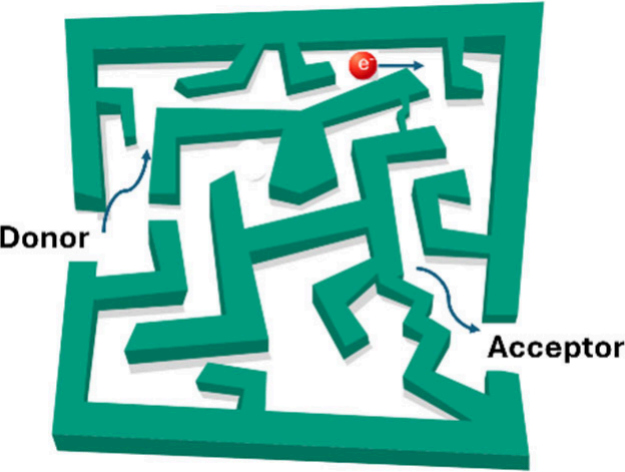

Electron transfer (eT) processes have garnered the attention of chemists and physicists for more than seven decades, and it is commonly believed that the essential features of the electron transfer mechanism are well understood—despite some open questions relating to the efficiency of long-range eT in some systems and temperature effects that are difficult to reconcile with the existing theories. The chiral induced spin selectivity (CISS) effect, which has been studied experimentally since 1999, demonstrates that eT through chiral systems depends on the electron’s spin. Attempts to explain the CISS effect by adding spin–orbit coupling to the existing eT theories fails to reproduce the experimental results quantitatively, and it has become evident that the theory for explaining CISS must consider electron–vibration and/or electron–electron interactions. In this Perspective we identify some features of the CISS effect that imply that we should reconsider and refine the Marcus–Levich–Jortner mechanistic description for eT processes, especially for nonlinear systems and in the case of long-range eT.

One of the profound conceptual achievements in Chemistry during the latter part of the twentieth century was the development of Marcus theory for electron transfer (eT) reactions–one of the most important and ubiquitous classes of chemical reactions.^1^ This framework assumes a time scale separation between nuclear and electron dynamics (Born–Oppenheimer and Condon approximations); and the electron motion is often treated through an effective one electron (orbital) picture, *vide infra*. The discovery of the chiral induced spin selectivity (CISS) effect, which manifests as spin-dependent electron transfer (and electron transmission) through chiral molecules^2,3^ has revealed new aspects of eT that challenge our current understanding. Upon its discovery, the theoretical approaches that were explored to explain the CISS effect were based on single electron models.^4^ For example, consider that an electron follows a helical path through a molecule and that the centripetal force acting on the electron, while moving along the curved path, generates an effective magnetic field in the rest frame of the electron. This effective field interacts with the magnetic moment of the electron, stabilizing one spin and destabilizing the other. Clearly, the spin degree of freedom in such a system is coupled to the orbital angular momentum; and when one refers to “spin polarization” one means the spin as measured by its projection from the total angular momentum of the electron. Despite qualitative agreement with the experiments, such a model fails to reproduce the large spin-dependent effects that are experimentally observed unless large spin–orbit coupling (SOC) values, much larger than those extracted from spectroscopic measurements, are invoked. Recent work suggests that many body effects can give rise to large effective SOC, *vide infra*. In this perspective, we consider our growing knowledge about CISS and its implications for an improved understanding of electron transfer reactions.

## Background on Electron Transfer

Investigations into redox reaction kinetics began in earnest after World War II with the development of Marcus’ classical eT theory and the ability to test those ideas with self-exchange reactions.^5^ These studies improved our understanding of solvent effects on eT reaction kinetics and opened the way to more incisive experiments that could begin testing important features associated with inner sphere reorganization energies (the role of nuclear vibrations) and how the eT rates are affected by the strength of the interaction between reactant and product electronic states (nonadiabatic to adiabatic dynamics). These efforts led to the Marcus-Levich-Jortner theory which provides the foundation for our current understanding of molecular eT, as well as electrochemical eT.^6^ This picture implies that in many cases the eT rate *k*_*eT*_ can be written in a compact form as
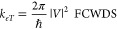
1where *V* is the electronic coupling and FCWDS is a Franck–Condon weighted density of states. This formulation assumes that the electronic and nuclear motions are separable (Born–Oppenheimer approximation) and uses a nonadiabatic representation for the reactant and product states. The electronic Hamiltonian is not diagonal in this representation and *V* describes the electronic coupling between the reactant and product states. Typically, workers assume that *V* does not depend on the nuclear coordinates (Condon approximation), at least over the relevant region of phase space. Nuclear degrees of freedom (FCWDS) often dominate the eT reaction kinetics and effects of solvent polarization,^7^ vibrational dynamics,^8^ and solvent friction^9,10^ on the reaction dynamics are known to be important in many cases; however, the basic Born–Oppenheimer (BO) picture is believed to hold. The nature of the electronic coupling *V* and its quantum mechanical description has been widely studied theoretically,^11^ but is challenging to study experimentally.

Although the adiabatic (or strong coupling) description was in vogue during the early investigations,^5^ the development of a quantum mechanical description for eT, with its origins in the theory of nonradiative transitions,^6^ has led to the nonadiabatic picture being dominant since the 1980s. The transition between the adiabatic and nonadiabatic descriptions is commonly described through a Landau–Zener treatment,^12^ and studies of electron transfer and electron transport in chiral molecules, and materials, span both regimes. For electron donor and acceptor moieties in physical contact (overlapping electron densities) direct electron exchange contributes strongly to *V*, however electron transfer reactions are unique in that they do not require direct contact between the reactants. For long distance electron transfer, the weak electronic coupling *V* is often described through a superexchange mechanism^13−15^ – a perturbation theory treatment involving negative anion (or cation) states of the atoms (or molecular subunits) that lie between the electron donor and acceptor moieties. In any case, the eT is treated as an effective single electron process and electron correlation effects are commonly neglected (or not included explicitly). Moreover, the electronic coupling is typically found through variational methods that evaluate the energy splitting (tunneling splitting) between donor and acceptor states, which arises from electronic interactions mediated by direct exchange or superexchange, and do not consider electron dynamics explicitly.

The established description of eT focuses almost solely on energetics and uses a transition state theory (quasi-equilibrium) approach for describing the rate constant. Although the initial reactant electronic state depends on nuclear degrees of freedom and its energy changes with solvent polarization, the electronic transition itself is considered to occur rapidly enough that the nuclear degrees of freedom are “frozen”. Moreover, the electron transfer (electronic transition probability) is controlled by the value of *V* which is found by energy considerations and neglects other dynamical variables, such as angular momentum; however, it does consider symmetry constraints imposed on the wave functions and/or the spin states.

## Is CISS Consistent with the Accepted eT Picture?

The connection between the electron spin (its intrinsic angular momentum) and a molecule’s chiral symmetry is not an explicit feature of eT theory, however chiral symmetry effects can be incorporated into calculations of the electronic coupling *V*. For spin to manifest in the exchange coupling between the donor and acceptor units, spin–orbit coupling must contribute to *V*. Numerous theoretical efforts along these lines have used helical or curved molecular structures in which the superexchange mechanism gives rise to an effective spin–orbit coupling^16,17^ or by invoking spin–orbit coupling directly.^18,19^ These works show that the spin–orbit coupling (SOC) one expects, based on spectroscopic measurements, is too small by several orders of magnitude from what is required to reproduce the experimental results. As an aside, we note that many experiments examine chiral molecules adsorbed to an electrode and may have contributions to the SOC from the substrate; and although the contribution of the substrate may be important in some cases, spin-dependent eT has been reported for molecules not adsorbed to surfaces^20^ so that this mechanism does not provide a universal explanation.

Recent work shows that spin-selective transport in chiral materials (conjugated polymers, semiconductors, and metals) can proceed over very long distances, exceeding hundreds of nanometers and extending to micron and even mm lengths.^21^ The mechanism for such long-range electron transfer often involves electron hopping, quasiparticle transport (polarons or solitons), or band transport. Note that long-range hopping is a slow process and relies on thermal activation,^22^ so that spin dephasing processes will contribute strongly. It is difficult to envision that spin-polarized electron currents will survive such processes unless some sort of spin polarizing process proceeds through the system as well. Some studies aimed at examining the spin coherence length in chiral systems exist,^23^ but more work along these lines is required before the implications are clear. These facts suggest that the origins of CISS are not rooted in a specific conduction mechanism.

Experiments into spin-dependent charge displacements and electron transmission in chiral systems (CISS) display other interesting features that bear on the nature of eT processes.

- CISS is not a linear effect, as expected for a process involving an energy barrier. Moreover, the spin-filtered electron current often depends nonlinearly on the applied voltage. This explains why the Onsager principle for experiments performed with two metal contacts need not hold for CISS measurements of molecules in molecular junctions or monolayer films.^4^
- In common magnetoresistance studies, when electrons are conducted between two ferromagnetic electrodes with an achiral layer in between, the preferred spin for positive voltage is the same as that for the negative voltage in the lab frame, but changes in the electron reference (from parallel to antiparallel to the electron flow). For a chiral material between a normal electrode and a ferromagnetic electrode, the preferred spin changes sign with a change in voltage sign in the lab frame, but it changes if the material’s handedness changes.^24,25^ That is, the spin selection is enantiospecific and can be controlled by selection of the enantiomer (or enantiomorph).
- Numerous studies^3^ show that the spin polarization observed for electron currents is correlated with a chiral material’s molar ellipticity, i.e., its circular dichroism strength, in which the light driven electron motion, or current, generates a transition magnetic moment leading to differential absorption of circular polarized light.^26^ These findings suggest that the anisotropic magneto-electronic polarizability may be a predictor for the strength of the CISS effect. Given the role of polarons for conduction in organic electronic devices, this may not be so surprising; yet, it is rarely considered for electron transfer through molecules.
- CISS phenomena manifest at room temperature, with spin selectivity as high as 100% reported in some cases.^27,28^ Thus, CISS is not caused by energy splittings associated with Zeeman effects, Δ*E*_Zeeman_, which are much smaller than *kT* at room temperature.

## What Does CISS Imply Is Missing from Conventional eT Theory?

The conventional eT picture places a strong emphasis on energetics and largely neglects the conservation of electron angular momentum (as well as linear momentum) in the eT dynamics. As an electron moves through a chiral potential, it will experience a force moving it along a curved path. Thus, it must exchange momentum with the other electrons and nuclei. Recent theoretical studies have pointed to the importance of angular momentum considerations in eT.^29^ Theoretical models that include many body effects, via electron–phonon interactions and/or electron–electron coupling, yield spin polarization of electron currents that are comparable to those observed experimentally.^17,30^ This finding implies that such interactions can play an important role in eT.

- *Electron–phonon coupling*: Recent work implies that the coupling of chiral phonons, defined as nuclear vibrations which carry angular momentum, to the electron’s spin angular momentum provides a microscopic explanation for well-known macroscopic phenomena, such as the Einstein-de Haas effect and its inverse, the Barnett effect.^31^ That is, the conservation of angular momentum manifests in a coupling between magnetization and mechanical degrees of freedom. How might such coupling manifest for CISS? Consider the chiral path of a moving electron charge that drives chiral nuclear displacements (phonons, vibrations, ···) through electrostatic interactions. In turn, these chiral charge displacements of the nuclei generate local currents and magnetic moments in the medium. Such a “chiral polaron” could propagate through the molecule (or material) and give rise to large spin selectivity.^32^ For an achiral molecule, chiral phonons do not manifest or they comprise a degenerate pair of vibrations which can display either handedness, thus leading to no spin preference. Whether or not this particular mechanism operates in chiral materials, it seems clear from model studies that the Born–Oppenheimer (BO) approximation is too limiting for describing electron transfer through chiral systems and non-BO effects may lead to large effective SOCs.
- *Electron–electron coupling*: An electron moving through a chiral molecule possesses an angular momentum comprising orbital and spin components, and as it propagates the total angular momentum of the system (electron and molecule) must be conserved. Experiments imply that the electron spin is coupled to the molecular frame of a chiral molecule. In fact, an early current transfer model for CISS^33^ approached this issue but only partially conserved the electron’s momentum. How might electron–electron interactions affect CISS? Consider long-range electron transfer through a molecule by way of a superexchange mechanism, in which the superexchange pathways operate through the bridging atoms but the incident electron does not necessarily pass through. That is, when an electron is injected in one end of the system, another electron is ejected from the other end.^34^ In this picture, the spin selectivity (and conservation of spin angular momentum) could arise from the incident electron interacting primarily with a subpopulation of the spins in the electron bath through Pauli exclusion, and the orbital angular momentum of the incident electron partial wave which transmits through the molecule is constrained by the chiral structure (e.g., helical sense). This picture implies that the molecule’s magneto-electronic polarizability tensor, which can be modeled by way of superexchange couplings, is important for understanding spin-selective eT. While this mechanistic picture still places a strong emphasis on energetics, it does so with spin and orbital constraints.

While the specific mechanistic scenarios discussed above may prove to be too simplified, the major conclusion remains that we need to move our description of eT beyond an effective one-electron picture and to consider non-BO effects explicitly. This is especially important for eT through curved systems.

Through studies into the CISS effect, in which the spin is an additional property that is probed for electron transfer through chiral systems, it has become clear that our current understanding of eT is incomplete. Important topics to consider for an improved description are-

### Angular Momentum Conservation

For an electron following a nonlinear path, momentum exchange with the rest of the molecule must be included and necessarily involves electron–electron and electron–phonon interactions. If the system through which the electron passes has bands, so that the bound electrons are extensively delocalized, then momentum can be exchanged between the transferring electron and the bound electrons very efficiently. In contrast, a system with little electron delocalization (e.g., a protein) may require low energy vibrations, which contain angular momentum, to enable the spin selective eT.

### Spin–Orbit Coupling (SOC)

Introducing electron–nuclear interactions and electron–electron interactions will change our perspective on SOC in chemistry.^35,36^ As was discussed above for electron–nuclear coupling in chiral materials, the electron motion can drive orbital nuclear motion (chiral polaron motion) giving rise to new types of SOC that chemists do not usually consider. This form of SOC would apply to itinerant (or transiting) electrons and cannot be extracted by electronic spectroscopy of eigenstates.

### Magneto-electronic Polarizability

The spin-dependent electronic polarization of a chiral molecule (or chiral medium), due to a moving electron, ought to be considered for understanding eT over long distances. That is, long-range spin and charge transport need not proceed by an injected electron from an electron donor (or source electrode) transiting through the molecule (or medium) to the acceptor (or drain electrode), rather the charge and spin reorganization that an injected electron produces can induce the release of some other electron to the acceptor. The charge reorganization is subject to important quantum mechanical constraints, such as indistinguishability and Pauli exclusion, and dynamical constraints, such as angular momentum conservation. By way of example, recent work shows that spin-induced charge reorganization can dramatically affect reactivity of proteins, even when the molecular moiety causing the spin-dependent electron cloud reorganization is attached far from the reaction site.^37,38^ The coupling mechanism and charge reorganization ought to be amenable to direct exchange and superexchange modeling;^33^ however, experiments suggest that the transmission of spin-polarized charge currents and the transmission of pure spin currents may manifest somewhat differently.^39^

### CISS Can Break Entanglement

Experiments have shown that a singlet state, in which two electrons are entangled, can survive eT processes in a linear system.^40^ In a chiral system, however, the entanglement should break, at least partially, due to the CISS effect; i.e., a preference exists for transferring one spin over the other. This is also the case when a chiral molecule is charge polarized.^41^ These finding imply that spin exchange interactions are likely important when considering eT in chiral systems. The fact that charge polarization and charge transfer occur for many molecules that are not completely symmetric suggests that spin exchange interactions should be considered for eT in those cases also.

With this perspective article, we hope to initiate a renewed discussion about the electronic coupling mechanism for eT reactions, which is enlightened by studies into the CISS effect. As part of the angular momentum of the electron, the spin is affected by the angular and linear momenta in molecules and momentum conservation should be considered for describing the electron dynamics. Given its foundation in the Born–Oppenheimer approximation, Marcus-Levich-Jortner theory neglects these conservation laws. New approaches to eT theory, which include non-BO considerations,^42−44^ are being explored. It will be interesting to see how studies into the mechanism of CISS might affect and refine our understanding of eT reactions and whether they can provide answers to open questions like the temperature effects, the long-range transfer, and the role of vibrations.^34^
